# European pond turtle *(Emys orbicularis persica)* as a biomarker of environmental pollution in Golestan and Mazandaran provinces, Iran

**Published:** 2017-12-15

**Authors:** Somayeh Namroodi, Annalisa Zaccaroni, Hassan Rezaei, Seyyedeh Malihe Hosseini

**Affiliations:** 1 *Department of Environmental Science, Faculty of Environment and Fisheries, Gorgan University of Agricultural Sciences and Natural Resources, Gorgan, Iran;*; 2 *Department* *of* *Veterinary Public Health and Animal Pathology, Faculty of Veterinary Medicine, University of Bologna, Bologna, Italy;*; 3 *Msc student, Department of Environmental Science, Faculty of Environment and Fisheries, Gorgan University of Agricultural Sciences and Natural Resources, Gorgan, Iran.*

**Keywords:** European pond turtle, Heavy metal, Golestan, Mazandaran

## Abstract

Anthropogenic environmental changes are hypothesized as main reasons for animal species population declines. Heavy metals contamination is one of the worst threats to animals among human-caused threats. As most of the heavy metals bioaccumulate in organisms, analyzing concentrations of heavy metals in long living animals, such as turtles, would be very useful for biomonitoring of environmental quality. The European pond turtle is classified as a Near Threatened in the red list of International Union for Conservation of Nature. The objective of this study was to obtain information on heavy metals contamination in this species, as a sentinels, to evaluate the overall health of both the European pond turtles and their ecosystem in Golestan and Mazandaran provinces. Biological samples of 10 living and 15 dead European pond turtles were analyzed by atomic absorption spectrophotometer for Zn, Pb, Cu, and Cd contaminations. Highest concentration of Zn (202.6 ± 58.5 μg g^-1^), Cd (4.4 ± 1.3 μg g^-1^) and Cu (3.8 ± 1.7 μg g^-1^) was detected in livers and the highest accumulation of Pb (45.6 ±16.3 μg g^-1^) occurred in kidneys. Positive correlations were detected among Zn, Pb and Cd tissue concentrations and carapaces curve length. Heavy metal levels were higher in males than females. Heavy metals contamination of sampled turtles stood in high degree. However, there is clearly a need to evaluate heavy metals physiologic effects on European pond turtles.

## Introduction

The European pond turtle (*Emys orbicularis*), is a long-living freshwater species of turtles that has been mainly considered as a semi-aquatic, with 1000 to 4000 meters terrestrial movement. This species prefer to live in a variety of freshwater habitats, including ponds, lakes, streams, rivers and drainage canals. Species of *Emys orbicularis* also inhabit upland environments for feeding.^1^

The diet of European pond turtle changes with age but mostly they prefer fish, amphibians, tadpoles, worms, mollusks, crustaceans and aquatic insects, as well as foraging on plants, so it is located in carnivores animals group.^2^

Unlike what its public name indicates, the European pond turtle is not limited to Europe, but actually has a varied distribution that also comprises northern Africa, western and central Asia.^3^

Recent studies have revealed that population of this species have been declined in most countries even though they are widely distributed. In France, European pond turtle has been introduced as the most endangered reptile of the country and unfortunately, in Switzerland, the turtle became extinct at the beginning of the 20^th^ century.^4^ Generally speaking, the European pond turtle is classified as a Near Threatened in red list of the International Union for Conservation of Nature. Basic data on the status of the European pond turtle populations are extremely limited in Iran. It can be assumed that European pond turtle populations decline like in other species of turtles, is occurring also in Iran as a negative result of anthropogenic impacts including urban and agricultural development, habitat loss, fragmentation and road mortality.^5^

Heavy metals contamination is one the worst threats to animals among human-caused threats. Heavy metals are natural constituents of the earth's crust, with high persistence and accumulation in organisms, however, indiscriminate human activities have severely changed their geochemical cycles and biochemical balance.^6^

Although, heavy metals such as copper (Cu) and zinc (Zn) are essential for normal biological functioning, some of them such as mercury (Hg), lead (Pb) and cadmium (Cd) are biologically non-essential for all living organisms and are toxic even in low concentrations. These heavy metals are important metals for industrial applications.^7^ Prolonged exposure to both essential and non-essential heavy metals can cause deleterious health effects on plants, animals and humans. As most of the heavy metals bioaccumulate in organisms, analyzing concentrations of so-called elements in animals, especially long living ones, that are upper in the food chain, would be very useful for environmental quality biomonitoring. ^8^

Over the past four decades, numerous wildlife species have been used as sentinels to investigate the health of their relevant environments. While there has been significant attention devoted to assessing the levels of contaminants in birds, mammals and even fish, little attention has been dedicated to reptiles as long living animals.^8^

Golestan and Mazandaran provinces natural ecosystems, which are parts of the northern habitat of European pond turtle in Iran, suffer from the large-scale industrial and agricultural developments.^9^

European pond turtles can serve as sentinels for their respective ecosystems and information on heavy metals contamination of this species can be used to assess both the overall health of the European pond turtles and their ecosystem. ^10^

Also, such information can then be used to plan management strategies for sustaining these animals and their environment in the future.

The objective of the present study was to measure the concentration of essential and non-essential heavy metals in biological samples of European pond turtles from Golestan province and evaluate if the contaminant levels were elevated and might pose a risk to the health of the population turtles.

## Materials and Methods

Sampling was conducted during 2014-16, in Golestan (36° 50’ N, 54° 26’ E) and Mazandaran provinces (36° 61’ N, 53° 23’ E), Iran. After determining the turtles’ species, 10 European pond turtles were hand captured when moving across roads, ponds or foraging in wetlands. Also, 15 European pond turtles were found died very recently on roads near rivers or Caspian Sea beach. Hand held global positioning system was used to determine the location of each sample. Once the turtles were given a number, a physical examination was performed. The turtles’ curved carapace length (CCL) was recorded using a standard metric measuring tape, as suggested by Auffenberg.^11^

Turtles were deeply anesthetized by intramuscular injection of 120 mg kg^-1^ ketamine (Alfasan, Woerden, The Netherlands) and 1 mg kg^-1^ xylazine (Alfasan) combination and were biopsied. Tissue samples (liver, kidneys and carapace) were collected during necropsy, placed in Ziploc^®^ bags and frozen at – 20 ˚C until analysis. Scutes were separated randomly from different parts of the carapace, cleaned with ultrapure water and washed in an ultrasonic bath to remove algae and other residues. The samples were weighed and homogenized. Approximately 1.0 g of sample was transferred into Pyrex^®^ digestion vessels, mixed with concentrated nitric acid (Merck, Darmstadt, Germany) and perchloric acid (Merck), (1:1) and then transferred to a hot block digester (model S4; BEHR, Stuttgart, Germany). After acidic digestion, samples were analyzed by atomic absorption spectrophotometer using an air/acetylene flame (GBC, Sydney, Australia). Two replicates of each sample were analyzed.

For statistical analyses, SPSS (version 20; SPSS Inc., Chicago, ‎USA) and Excel (version 12.0; Microsoft, Redmond, USA) were used to analyze obtained results. Kolmogorov–Smirnov test was performed to evaluate normality of the data. As all results were normally distributed, differences of contaminant concentrations between turtles, heavy metals, tissues and sexuality were calculated by ANOVA test. Correlations between CCL and heavy metals concentration were surveyed using Pearson correlation coefficient.

## Results

A summary of biometric data was recorded as sex (male = 10, female = 15) and maen CCL (females = 205 mm, males = 164 mm). Mean concentrations of the heavy metals analyzed in the livers, kidneys and carapaces of European pond turtles are presented in Table 1 as micrograms of metal per wet weight (ww) of tissue samples (μg g^-1^). Significant differences in essential and nonessential heavy metals concentrations in analyzed tissues were found (Table 1). Considerably high Zn concentration was found in sampled European pond turtles, followed by Pb, Cd and Cu (Table 1). Mean hepatic concentrations of Zn, Cu and Cd were higher than other sampled tissues, while Pb levels were the highest in kidneys (Table 1). Body size significantly influenced bioaccumulation of surveyed heavy metals in sampled tissues and a significant positive correlation (Rs = 0.900; *p* < 0.001) was observed among Zn, Pb and Cd concentrations and CCL. In contrast, Cu concentrations were decreased with increasing carapace length (Table 2).

**Fig. 1:**
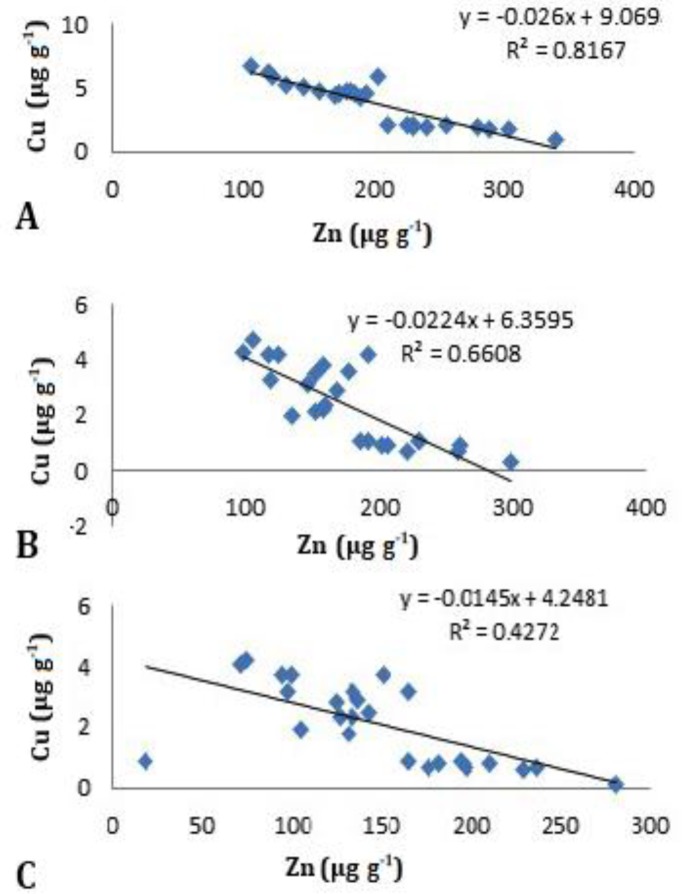
Correlation between zinc and copper concentrations based on scatter plot analysis. A= Liver, B= Kidney, C= Carapace.

Heavy metals concentration differed between genders and also contamination was significantly higher in males (Table 3). Negative correlation was observed between Zn and Cu accumulation in different organs (Fig. 1).

**Table 1:** Heavy metal levels (μg g^-1^) in different tissues. Data are presented as mean ± SD.

**Tissue**	**Lead**	**Zinc**	**Copper**	**Cadmium**
**Kidney**	45.6 ± 16.3	178.3 ± 50.7	2.3 ± 1.4	2.9 ± 1.1
**Liver**	42.5 ± 15.8	202.6 ± 58.5	3.8 ± 1.7	4.4 ± 1.3
**Carapace**	36.6 ± 15.3	147.4 ± 57.5	2.1 ± 1.3	1.8 ± 0.9

**Table 2:** Pearson correlation between tissue heavy metals concentration and carapace curve length (CCL).

	**Cu L**	**Cu K**	**Cu C**	**Cd L**	**Cd K**	**Cd C**	**Pb L**	**Pb K**	**Pb C**	**Zn L**	**Zn K**	**Zn C**
**CCL**	-0.666^**^	-0.677 ^**^	-0.696 ^**^	0.756 ^**^	0.735 ^**^	0.744 ^**^	0.670 ^**^	0.676 ^**^	0.629 ^**^	0.593 ^**^	0.562 ^**^	0.475^*^
**Correlation**	0.000	0.000	0.000	0.000	0.000	0.000	0.000	0.000	0.001	0.002	0.003	0.016

K = Kidney, L = Liver, C = Carapace.

‎* Correlation is significant at the 0.05 level (*p* ˂ 0.05);

** Correlation is significant at the 0.01 level (*p* ˂ 0.01).‎

**Table 3:** Mean heavy metals level (μg g^-1^) in male and female European pond turtles.

**Heavy metals ** **‎**	**Cu L **	**Cu K**	** Cu C**	**Cd L**	**Cd K**	**Cd C**	**Pb L**	**Pb K**	**Pb C**	**Zn L**	**Zn K**	**Zn C**
**Female**	2.9	1.7	1.5	2.1	3.3	1.1	30.3	33.1	24.4	160.8	142.0	113.8
**Male**	5.2	7.2	3.0	3.5	5.1	2.2	50.7	53.9	44.8	230.5	202.6	169.8

K = Kidney, L = Liver, C = Carapace.

## Discussion

As heavy metals mostly enter turtle’s body by consumption of foods, results of this study provided evidence for high presence of heavy metals in European pond turtle’s food chain in Golestan province.

There is limited data regarding heavy metals level in European pond turtles. For this study, as for other studies described in the scientific reports, the measured levels of contaminants in the European pond turtle’s samples were compared to the levels reported in the peer-reviewed scientific literatures for other species of turtles.

In this study, the highest concentration of tissue heavy metals was belonged to Zn followed by Pb, Cd and Cu. Different trend of heavy metal concentration (Zn, followed by Cu, Pb, Hg and Cd) has been reported in blood sample of Mediterranean Pond Turtle (*Mauremys leprosa*).^12^ This difference could be due to difference in surveyed biological samples between these two studies.

Cadmium is a non-essential metal with unknown biological functions.^13^ Results of survey on fresh water turtles (painted turtle (*Chrysemys picta*) and *Trachemys scripta) *provide evidence that environmentally relevant doses of Cd may affect gonadal developmental processes during embryonic and post-natal stages that may result in disruption of reproductive processes later in life.^14^ Major anthropogenic sources of Cd include smelter fumes and dusts, non-ferrous metal mining and refining, incineration and disposal of Cd containing waste and fossil fuels, fertilizers and municipal as well as sludge discharges.^15^

Cadmium values in sampled tissues are lower than those reported by Macêdo *et al*. in green sea turtles from cost of Bahia (18.8 ± 10.6 μg g^-1^),^16^ green sea turtle from Yaeyama Island (18.2 ± 9.7 μg g^-1^) and red eared slider turtle (*Trachemys scripta elegans*) from Kentucky^17^ and higher than capian pond turtles in surveys of Adel *et al*. (4.55± 1.32 dry weight)^18 ^and Yadollahvand *et al*. (4.29 ± 1.9)^19^ in North of Iran. However, it remains unidentified whether this difference is due to the exposure history, ontogenetic change and linked lower Cd consumption in the carnivorous life stages or an associated change in physiology/metallothionein production capacity of European pond turtle.^20^^,^^21^

 In comparison with other similar surveys, concentration in European pond turtles was almost similar to the average kidney Cd of adult and juvenile turtles from Brazil (18.8 ppm),^16^ Costa Rica (4.7 ppm ww),^22^ Turkey (1.9 ppm ww)^23^ and Mexico (1.6 ppm ww). ^24^

Cadmium levels were higher in liver tissues than carapace samples. This trend of accumulation has been reported in Caspian pond turtels by Adel *et al*. and Yadollahvand *et al*. too.^18^^,^^19^

Although, Kidney Cd levels were higher than livers and carapace samples which has been similarly reported in red eared slider turtle (*Trachemys scripta elegans*) from Kentucky.^17^ Actually, tissue Cd accumulation pattern in this study was in line with known Cd tendency to bind with metallothionein and accumulate preferentially in kidneys and liver of mammals and turtles ^22^^,^^25^^,^^26^

It has been suggested that such pattern in Cd accumulation reveals chronic exposure to Cd. Metallothionein is synthesized in liver, then transported in the bloodstream to the kidney, where it accumulates at higher levels than liver following long-term exposure. Following short-term exposure, both tissues contain similar concentrations, except for very high levels of exposure, when liver is higher in metallothionein.^22^^,^^27^

Lead, a non-beneficial and non-essential heavy metal, is a major environmental pollutant. Paint, cosmetics, human medicines, food supplements and petroleum-based fuels have been considered as sources of lead pollution. ^13^

In comparison with most of the related studies in turtles, mean concentration of Pb in sampled European pond turtles was located in high degree. Mean kidney Pb levels of 11.85 μg g^-1 ^ww and 6.6 μg g^-1 ^ww, have been reported in green and hawksbill sea turtles, respectively.^16,28^ Also, survey on Pb concentration in Caspian pond turtles by Adel *et al*. (35.46 ± 1.90 μg g^-1^dw) and Yaddolahvand et. al (32.41 ± 6.22 μg g^-1 ^dw) indicates a lower mean level of kidney Pb in this species. Similarly, Adel *et al*. and Yaddolahvand *et al* ., found the lowest level of Pb in carapaces of Caspian pond turtles.^18^^,^^19^ But, Overman *et al*. reported the hieghst levels of Pb in carapace tissues of sampled snapping turtles *(Chelydra serpentina)* in USA.^29^

Lead levels were significantly higher in kidney tissues compared with other analyzed samples. Results of similar studies have shown various pattern of Pb concentration in biological samples. Similarly, Yaddolahvand *et al*. detected higher Pb concentration in kidney tissues than liver tissues of Caspian pond turtles.^19^ On the contrary, Yu *et al*. found highest concentration of Pb in liver tissues of red eared slider turtle (*Trachemys scripta elegans*). With regard to these results, it could be assumed that Pb tissue tendency depends on the species.^17^

The health effects of Pb on European pond turtles are unidentified, but studies on mice and rats showed that high levels of Pb might be harmful to the health and longevity of animals.^30^

Zinc and copper, trace mineral nutrients, are located among the most common essential elements for organisms. They participate in small quantities in same chemical and physiological activities which affect animal’s growth, immunity and reproduction function. A large amount of Zn and Cu enters in the environment as a result of mining, purification of zinc, lead and cadmium ores, steel production and coal burning which result in Zn and Cu toxicities.^31^

Compared to the results of similar studies on semi-aquatic and fresh water turtles (Caspian pond turtles, snapping turtles and red eared slider turtle (*Trachemys scripta elegans*), Cu concentrations in sampled European pond turtles stay in higher degree.^17^^-^^19^^,^^32^

In this study, negative correlation was detected between tissue concentration of Cu and Zn. It has been documented that chronic exposure to Zn can cause decrease in absorption of Cu from the diet.^27^ Therefore, the observed negative correlation can highlight chronic exposure of European pond turtles to Zn in Golestan province. More physiological studies on this species are needed to better define the role and correlation between Zn and Cu levels in European pond turtles.

In the present study, biological samples of European pond turtles contained considerable levels of Zn, especially in livers. This result is in line with other studies performed in various turtle species, identifying the liver as a major site of Zn accumulation.^26^^,^^33^^,^^34^ A different trend was instead reported in Caspian pond turtles with highest Zn concentration in muscle samples.^19^

Considerably, high accumulation of Zn, Cd and Cu in the liver of European pond turtles was observed. It can be explained by the major role of liver in detoxification process of blood and binding of metallothioneins with heavy metals in this tissue. Actually, metallothionein play a vital role in trace element homeostasis and defense against trace element toxicity.^28^^,^^35^

Result of this study showed that despite variations in bioaccumulation of analyzed heavy metals, trace elements tend to have a proportionally similar tissue tropism, as already reported in fresh water turtles.^18^^,^^19^ Consequently, these factors can be considered as important factors to take into consideration when evaluating environmental pollution risks and designing future monitoring and restoration plans for organisms polluted by heavy metals.

Female European pond turtles had higher concentrations of tissue heavy metals than males at a given size. This pattern is opposite to some studies that suggest females should have lower heavy metals body burdens since they maternally transfer some of them, such as Hg to their eggs, thus providing females with an additional elimination pathway.^10^ In surveye of Martínez-López *et al*. on Mediterranean pond turtle (*Mauremys leprosa*), males presented higher levels than females, which explained by maternal transfer during egg formation.^12^ Although, Adel *et* *al*. and Bishop *et al*. reported that there was no significant difference in heavy metal concentration between genders in Caspian pond turtles and emydid turtles, respectively.^18^^,^^36^

It can be assumed that higher heavy metal concentrations present in females tissues may be due to sexual dimorphism in size and growth.^37^ Different species of turtles display a strong body size dimorphism, in which adult females may grow and also asymptote at a smaller size compared to males which rely on their size to contest and protect territories.^37^^,^^38^

Given this growth pattern, it is likely that females, though smaller, may be older and subsequently have been exposed for longer time to heavy metals with respect to males of similar size. Alternatively, gender may affect feeding rates and/or feeding ecology, but little is known about sex-specific differences in feeding ecology of European pond turtles. 

Body size significantly influenced heavy metal concentration in European pond turtles with Pb, Cd and Zn concentrations increasing with carapace length. There are several reports that indicate increase of heavy metals concentration such as Zn and Pb with age and body size.^12^^,^^39^^-^^41^ However, in survey of Adel *et al*. on Caspian pond turtle (*Mauremys caspica*) from the southern basin of Caspian Sea, no correlations were found in metals concentrations with size or weight.^18^

The Cu concentration pattern wasn’t in line with body size growth and higher concentration of Cu was detected in smaller European pond turtles. Similar trend in Cu accumulation was documented in Mediterranean pond turtle (*Mauremys leprosa*).^12^

As a long-living animal, larger European pond turtles cannot be surely older ones. However, positive correlation between heavy metals accumulation and body size growth shows heavy metal biomagnifying in tissues of European pond turtles.

Difference between results of this study with mentioned studies can be explained by different species-specific ecological strategies. Indeed, it has been proved that different dietary patterns in various turtle species can lead to different levels of heavy metals in their bodies.^42^

Also, sampling region, size and age of sampled animals can affect the heavy metals exposure in animals. Anan *et al*. suggested that differences in trace element concentrations cannot be solely explained by these factors and further studies are needed regarding dynamic interactions of turtles at different life stages in order to measure the trophic transfer of elements in these animals.^43^

Results of similar studies indicate that similar to vertebrates, tissue levels in turtles parallel the degree of environmental contamination.^40^^,^^44^^,^^45^

Particularly for metals and metalloids, however, it is difficult to establish what constitutes non-elevated, normal background levels in sampled European pond turtles due to the spatial variability of naturally occurring elements.

Overall, these comparisons suggest that European pond turtles from this study were exposed to elevated levels of heavy metals that resulted in tissue levels that mostly surpass those reported from other surveyed turtles from different places.

It has been accepted that sensitivity to heavy metals toxicity is species-specific, making it impossible to predict heavy metal toxic thresholds for sampled European pond turtles from the limited available data. However, obtained results suggested that heavy metals concentrations in European pond turtles were high enough to pose a possible risk of harmful effects not only to this species but also to the wild animals and human population that live in Golestan and Mazandaran provinces.

There is clearly a need to evaluate environmental pollutants and their physiologic effects in European pond turtles in an effort to determine if they have any negative impacts on these animals.

## References

[1] Ficetola GF, Bernardi FD (2006). Is the European pond turtle (Emys orbicularis) strictly ‎aquatic and carnivorous?. Amphib Reptil.

[2] Ottonello D, Salvidio S, Rosecchi E (2005). Feeding habits of the European pond terrapin ‎Emys orbicularis in Camargue (Rhône delta, Southern France). Amphib Repti.

[3] ‎Schneeweiss N, Steinhauer C (1998). Habitat use and migrations of a remnant population of the ‎European pond turtle, Emys oorbicularis (Linnaeus, 1758), depending on landscape structures in ‎Brandenburg, Germany. Mertensiella.

[4] ‎Fritz U, Wischuf T (1997). To the systematics of West-Asian-Southeast European turtles (genus Mauremys) (Reptilia: Testudines: Bataguridae)[German]. Zoologische ‎Abhandlungen-Staatliches Museum Fur Tierkunde In Dresden.

[5] ‎Daszak P, Cunningham A, Hyatt A (2001). Anthropogenic environmental change and the ‎emergence of infectious diseases in wildlife. Acta Tropica.

[6] ‎Godley BJ, Thompson DR, Furness RW (1999). Do heavy metal concentrations pose a threat ‎to marine turtles from the Mediterranean Sea?. Marine Poll Bull.

[7] ‎Flora S, Mittal M, Mehta A (2008). Heavy metal induced oxidative stress & its possible reversal ‎by chelation therapy. Indian J Med Res.

[8] ‎Sepe A, Ciaralli L, Ciprotti M (2003). Determination of cadmium, chromium, lead and vanadium in six fish species ‎from the Adriatic Sea. Food Addit Contam.

[9] ‎Kami HG, Hojati V, Pashaee Rad SH (2006). A biological study of the European pond turtle, Emys orbicularis persica, ‎and the Caspian pond turtle, Mauremys caspica caspica, in the Golestan and Mazandaran ‎provinces of Iran. Zool Middle East.

[10] ‎Meyers-Schöne L, Walton BT (1994). Turtles as monitors of chemical contaminants in the ‎environment. Rev Environ Contam Toxicol.

[11] ‎Auffenberg W, Iverson J, Harless M, ‎Morlock H (1979). Demography of terrestrial turtles. Turtles: Perspectives and research.

[12] ‎Martínez-López E, Gómez-Ramírez P, Espín S (2017). Influence of a former mining area in the heavy metals ‎concentrations in blood of free-living Mediterranean pond turtles (Mauremys leprosa). ‎ Bull Environ Contam Toxicol.

[13] Stohs S, Bagchi D (1995). Oxidative mechanisms in the toxicity of metal ions. Free Radic Biol Med.

[14] Kitana N, Callard IP (2008). Effect of cadmium on gonadal development in freshwater turtle ‎‎(Trachemys scripta, Chrysemys picta) embryos. J Environ Sci Health A.

[15] Eisler R (1987). Polycyclic aromatic hydrocarbon hazards to fish, wildlife, and invertebrates: A ‎synoptic review. Contaminant Hazard Reviews Report.

[16] de Macêdo GR, Tarantino TB, Barbosa IS (2015). Trace elements distribution in hawksbill turtle (Eretmochelys ‎imbricata) and green turtle (Chelonia mydas) tissues on the northern coast of Bahia, Brazil. ‎Marine Poll Bull.

[17] ‎Yu S, Halbrook RS, Sparling DW Metal accumulation and evaluation of effects in a fresh water turtle. ‎Ecotoxicology.

[18] ‎Adel M, Saravi HN, Dadar M Mercury, lead, and cadmium in tissues of the Caspian pond turtle (Mauremys ‎caspica) from the southern basin of Caspian Sea. Environ Sci Pollut Res.

[19] ‎Yadollahvand R, Kami HG, Mashroofeh A (2014). Assessment trace elements concentrations in tissues in Caspian pond ‎turtle (Mauremys caspica) from Golestan province, Iran. Ecotoxicol Environ Saf.

[20] ‎Laurent S, Delphine F, Marc P (2008). Magnetic iron oxide nanoparticles: synthesis, stabilization, vectorization, ‎physicochemical characterizations, and biological applications. Chem Rev.

[21] ‎Sakai H, Saeki K, Ichihashi H (2000). Growth-related changes in heavy metal accumulation in green turtle ‎‎(Chelonia mydas) from Yaeyama Islands, Okinawa, Japan. Arch Environ Contam Toxicol.

[22] ‎Andreani G, Santoro M, Cottignoli S (2008). Metal distribution and metallothionein in loggerhead (Carettacaretta) and ‎green (Cheloniamydas) sea turtles. Sci Total Environ.

[23] ‎Kaska Y, Celik A, Bag H (2004). Heavy metal monitoring in stranded sea turtles along the Mediterranean ‎coast of Turkey. Fresen Environ Bull.

[24] ‎Talavera-Saenz A, Gardner SC, Riosmena Rodriquez R (2007). Metal profiles used as environmental markers of green turtle ‎‎(Cheloniamydas) foraging resources. Sci Total Environ.

[25] ‎Agusa T, Takagi K, Kubota R (2008). Specific accumulation of arsenic compounds in green turtles (Chelonia ‎mydas) and hawksbill turtles (Eretmochelys imbricata) from Ishigaki Island, Japan. ‎ Environ Pollut.

[26] Storelli M, Storelli A, D'Addabbo R (2005). Trace elements in loggerhead turtles (Caretta caretta) from the eastern ‎Mediterranean Sea: Overview and evaluation. Environ Pollut.

[27] U.S. Department of Health and Human Services (1997). Toxicological Profile for Cadmium (Draft for Public Comment).

[28] Barbieri E (2009). Concentration of heavy metals in tissues of green turtles (Chelonia mydas) ‎sampled in the Cananéia estuary, Brazil. Braz J Oceanogr.

[29] Overmann SR, Krajicek JJ (1995). Snapping turtles (Chelydra serpentina) as biomonitors of ‎lead contamination of the Big River in Missouri's old lead belt. Environ Toxicol Chem.

[30] ‎Kaur S (1988). Lead in the scales of cobras and wall lizards from rural and urban areas of Punjab, ‎India. Sci Total Environ.

[31] ‎Luza SC, Speisky HC (1996). Liver copper storage and transport during development: ‎implications for cytotoxicity. Am J Clin Nutr.

[32] ‎Albers P, Sileo L, Mulhern B (1986). Effects of environmental contaminants on snapping ‎turtles of a tidal wetland. Arch Environ Contam Toxicol.

[33] ‎García-Fernández AJ, Ramirez PJ, Lopez EM (2009). Heavy metals in tissues from loggerhead turtles (Caretta ‎caretta) from the southwestern Mediterranean (Spain). Ecotoxicol Environ Saf.

[34] ‎Torrent A, González-Díaz OM, Monagas P (2004). Tissue distribution of metals in loggerhead turtles (Caretta caretta) stranded ‎in the Canary Islands, Spain. Marine Poll Bull.

[35] ‎Mashroofeh A, Bakhtiari AR, Pourkazemi M (2013). Bioaccumulation of Cd, Pb and Zn in the edible and inedible tissues of ‎three sturgeon species in the Iranian coastline of the Caspian Sea. Chemosphere.

[36] ‎Bishop BE, Savitzky BA, Abdel-Fattah T (2010). Lead bioaccumulation in emydid turtles of ‎an urban lake and its relationship to shell disease. Ecotoxicol Environ Saf.

[37] ‎Christiansen JL, Burken RR (1979). Growth and maturity of the snapping turtle (Chelydra ‎serpentina) in Iowa. Herpetologica.

[38] Galbraith DA, Chandler MW, Brooks RJ (1987). The fine structure of home ranges of male ‎Chelydra serpentina: are snapping turtles territorial?. Can J Zool.

[39] ‎Bergeron CM, Hopkins WA, Bodinof CM (2011). Counterbalancing effects of maternal mercury exposure during ‎different stages of early ontogeny in American toads. Sci Total Environ.

[40] Grillitsch B, Schiesari L, Sparling DW, Linder G, Bishop CA (2010). The Ecotoxicology of Metals in Reptiles. Ecotoxicology of ‎amphibians and reptiles Boca Raton.

[41] ‎Turnquist MA, Driscoll CT, Schulz KL (2011). Mercury concentrations in snapping turtles (Chelydra serpentina) ‎correlate with environmental and landscape characteristics. Ecotoxicol.

[42] ‎Bjorndal KA, Lutz PL, Musick JA (1997). Foraging ecology and nutrition of sea turtles. The biology of sea turtles. Boca Raton.

[43] ‎ Anan Y, Kunito T, Watanabe r (2001). Trace element accumulation in hawksbill turtles (Eretmochelys imbricata) and ‎green turtles (Cheloniamydas) from Yaeyama Islands, Japan. Environ Toxicol Chem.

[44] ‎Day RD, Steven JC, Paul R (2005). Monitoring mercury in the loggerhead sea turtle, Caretta caretta. ‎ Environ Sci Technol.

[45] ‎ Hermanussen S, Limpus C, Papke O (2006). Foraging Habitat Contamination Influences Green Sea Turtle PCDD/F Exposure. In proceedings: 26th International Symposium on Halogenated Persistent Organic Pollutants.

